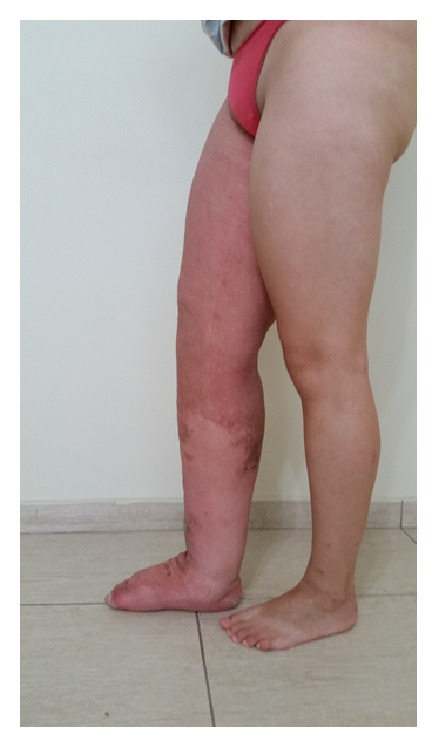# Lymphedema in Klippel-Trenaunay Syndrome: Is It Possible to Normalize?

**DOI:** 10.1155/2016/5230634

**Published:** 2016-07-26

**Authors:** Jose Maria Pereira de Godoy, Angela Río, Paloma Domingo Garcia, Maria de Fatima Guerreiro Godoy

**Affiliations:** ^1^Cardiology and Cardiovascular Surgery Department, The Medicine School in São José do Rio Preto (FAMERP) and CNPq (National Council for Research and Development), São José do Rio Preto, SP, Brazil; ^2^Universidad Europea of Madrid, Madrid, Spain; ^3^Research Group of Godoy Clinic, Sao Jose do Rio Preto, SP, Brazil; ^4^Medicine School in São José do Rio Preto (FAMERP) and Research Group of the Godoy Clinic, Sao Jose do Rio Preto, SP, Brazil

## Abstract

The aim of this study is to report the results of intensive therapy of lymphedema associated with Klippel-Trenaunay syndrome. A 24-year-old female patient reported that her family had observed edema in her right leg and port wine stains from birth. For ten years, they consulted with different specialists in the region but the prognosis did not change and no specific treatment was found. In 2014, at the age of 24, with massive lymphedema, a leg ulcer, and recurrent infections, she started treatment at the Clínica Godoy in São José do Rio Preto. She was evaluated by clinical history, physical examination, water displacement volumetry, and bioimpedance. Intensive therapy (8 hours daily) was proposed using Manual Lymphatic Therapy (Godoy & Godoy), Cervical Stimulation Therapy, Mechanical Lymphatic Therapy, a grosgrain stocking adjusted several times a day, and the use of Unna boot in the region of the ulcer. The volume of edema was reduced by about 44% within the first week with further reductions in the following weeks and healing of the ulcer. Subsequently, it was possible to control and maintain the reduction in swelling with less intense treatment. It is possible to reduce and maintain the treatment results of lymphedema associated with Klippel-Trenaunay syndrome.

## 1. Introduction

Klippel-Trenaunay syndrome is congenital angiodysplasia characterized by venous and lymphatic malformations, hypertrophy of the bone, and soft tissues, which, in most cases, affect just one extremity [1]. The lesions are present at birth, but often a port wine stain, a capillary lymphatic malformation, is the only visible sign; about 75% of patients manifest symptoms before the age of 10 years [2]. The deep venous system may be hypoplastic or absent and so carriers have the risk of developing infections, deep venous thrombosis, superficial thrombophlebitis, and pulmonary thromboembolism.

The differential diagnosis includes Chuvash polycythemia caused by a von Hippel-Lindau gene mutation resulting in a problem with oxygen sensing and Parkes-Weber syndrome characterized by capillary malformations with underlying arteriovenous malformations and hemihypertrophy (including bony overgrowth).

A precise diagnosis is clinically very important as these conditions are managed differently, and the differential diagnosis is rather difficult during childhood.

Although there are several hypotheses, the origin of this syndrome remains unknown. One study that evaluated 66 patients with Klippel-Trenaunay syndrome showed that 45 patients (74%) had predominantly venous defects, four (6%) had predominantly lymphatic defects, and 12 (20%) had mixed vascular defects. Extratruncal lymphatic malformations were found in 13 patients (21%) and truncal lymphatic malformations in 17 (28%) [3]. Thus, lymphedema may be present from early childhood.

Lymphedema is a chronic medical condition in which there is an accumulation of water, salts, electrolytes, high molecular weight proteins, and other elements in the interstitial space resulting in dynamic or mechanical changes of the lymphatic system. This condition causes a reduction in the functional and immunological capacity and morphological changes of the system and weight gain [4]. Conservative treatment of lymphedema is recommended in these cases using a combination of therapies [5]. The association of therapies recommended for the treatment of lymphedema includes manual lymph drainage, compression therapy, exercise, myolymphokinetic activities, and hygiene care [6, 7].

In recent years, new options have been developed such as mechanical lymph drainage with devices that produce passive muscle activities [8]. Intensive forms of lymphedema treatment have been reported with the possibility of rapid control of edema and maintenance of treatment results [8]. The aim of this case study is to report on the evolution of intensive treatment in lymphedema associated with Klippel-Trenaunay syndrome.

## 2. Case Report

We report the case of a 24-year-old female patient who reported that her family noticed swelling of the right leg and the presence of port wine stains at birth. At four months, her parents consulted with a specialist who arrived at a diagnosis of Klippel-Trenaunay syndrome and told the parents that the prognosis was bad with no prospect of normal motor development. However, the patient's motor performance developed normally and she began to walk at the age of one. For ten years, the parents consulted several specialists in the area where they live who did not change the prognosis and indicated no specific treatment. At 15 years of age, an ulcer appeared in the dorsomedial region of the leg for which treatment was sought in a health clinic where the wound was dressed but it did not heal. Two years later, the patient had an outbreak of erysipelas and was hospitalized for 28 days. This was followed by further hospitalization for outbreaks of erysipelas, which resulted in worsening of the edema and ulceration. At 18, the patient presented much exudate from the open ulcer and as the edema had worsened, she consulted with a vascular surgeon in Porto Alegre in her home state who, after an examination, indicated lymphedema treatment in a vascular service in São Paulo. In 2013, at the age of 24 and with massive lymphedema, an ulcer, and repeated infections, she decided to ask for help from the media and was told of a specialist service in São José do Rio Preto.

In the Clínica Godoy, her clinical history was evaluated and she underwent a physical examination with volumetry using the water displacement technique and bioimpedance. The diagnosis of Klippel-Trenaunay syndrome was confirmed clinically. The patient reported that the different specialists she consulted had made several additional tests but she did not present the results and did not provide any details about the treatments performed. The aim of the proposed therapy is to normalize edema and thus bioimpedance is critical to monitor the condition of lymphedema. Intensive treatment of eight hours/day was proposed using Manual Lymphatic Therapy (Godoy & Godoy), Cervical Stimulation Therapy, Mechanical Lymphatic Therapy (RAGodoy), a grosgrain stocking adjusted several times daily, and an Unna's boot in the region of the ulcer.

**Figure 1:**
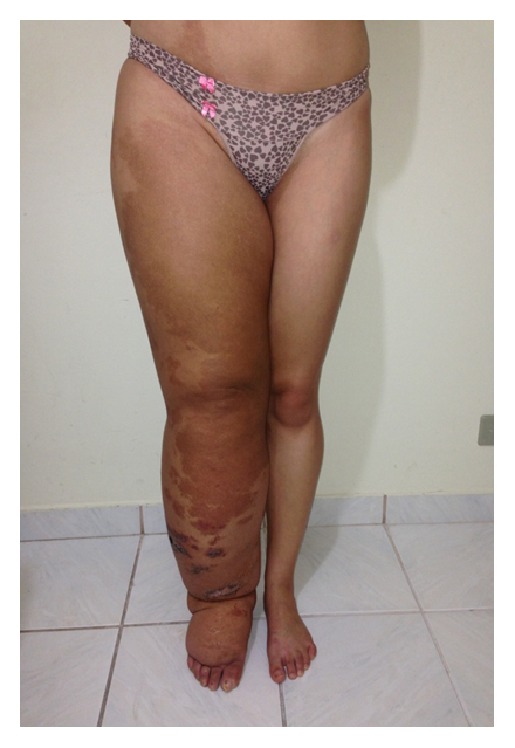


**Figure 2:**
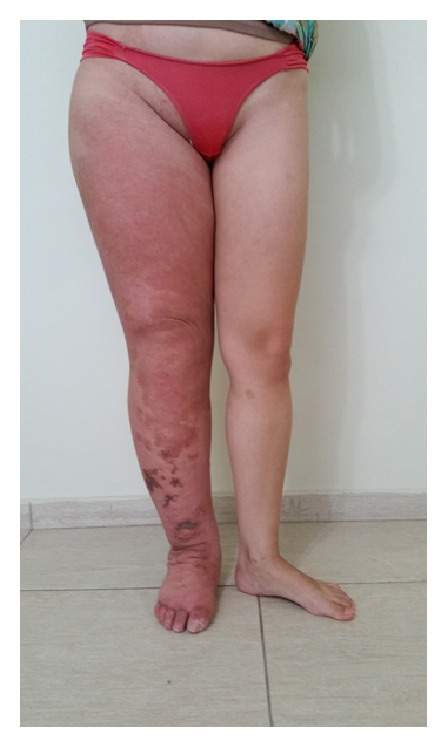


The volume of the lymphedematous lower leg, as assessed using the water displacement technique, was 8.626 L as opposed to 2.627 L for the normal leg thereby giving a difference of 5.999 L (Figure 1). In the first week, the volume was reduced to 5.971 L, the equivalent of 44% of the volume of lymphedema. Two other weeks of intensive treatment were conducted, once per year, and the volume was reduced to 4.334 L (Figures 2 and 3). By bioimpedance (InBody S10) the lymphedematous leg had a volume of 14.23 L and the normal leg 5.80 L giving a difference of 8.43 L. The most recent assessment showed that the lymphedematous leg had reduced to 6.63 L and the normal leg to 5.16 L. The extracellular water ratio from the start of treatment (0.434) increased to 0.396, with the normal ratio being 0.39. With treatment, an increase in fluid content of the upper extremities was observed from 1.29 L to 1.39 L for the right arm and from 1.28 L to 1.42 L for the left. The fluid content of the chest also increased from 13.2 L to 13.9 L.

## 3. Discussion

Herein we report on a case of elephantiasis associated with Klippel-Trenaunay syndrome in a 24-year-old patient with an untreated ulcer and recurrent erysipelas in which an intensive approach was used to treat the lymphedema. This type of patient management has not been reported in the literature with respect to Klippel-Trenaunay syndrome involving the lymphatic system.

The first aspects to be considered are the difficulty of the patient to find effective therapy and the quick progression to elephantiasis. The frequent episodes of erysipelas were an aggravating factor that required several interventions. The difficulty in healing of the ulcer was another aspect that impaired the quality of life of the patient.

Intensive outpatient treatment of lymphedema reduces the volume of edema by about 50% within five days [9]; this patient lost 44% of the excess limb volume. This fact changed the whole evolution of the clinical condition in a short period and demonstrates the credibility of the approach for a patient that had sought clinical treatment for years. The main goals of the techniques employed in the Godoy & Godoy method are to facilitate the passage of macromolecules from the interstitial space to the lymphatic capillaries followed by lymph drainage.

This approach uses an association of techniques where Mechanical Lymphatic Therapy (RAGodoy) [8] is performed every day of the therapeutic program (8 hours/day), Manual Lymphatic Therapy is employed for about one hour daily, and cervical stimulation is performed for about 15 minutes/day. However, the association of a constraint mechanism, such as compression stockings or bandages, is of fundamental importance because of their synergistic effect in reducing edema. The main constraint mechanism used in the treatment of this patient was a custom-made grosgrain stocking, which has important characteristics such as good working and resting pressures. Another detail is on the manual lymph drainage technique used (Manual Lymphatic Therapy), which is based on manual compression followed by linear displacement to the corresponding lymph node.

With treatment, the increase of fluids in normal body regions is explained by the mobilization of macromolecules with their redistribution. The loss of large volumes in weight is due to water loss; however, the macromolecules are not eliminated but rather redistributed to the upper limbs and chest body [10].

Klippel-Trenaunay syndrome is a congenital malformation that may involve arterial, venous, and lymphatic vessels leading to hypertrophy with or without edema. In this patient, the most important aspect was the involvement of the lymphatic system, where lymph drainage produced a decrease in the limb volume. The total reduction of edema did not normalize the limb size, but only with the hypertrophy component, and arterial and venous vascular changes remain. The use of a stocking in lymphedema also diminishes the effects of venous changes.

Another important factor was the improvement of the psychological aspects that allowed improvements in the patient's body image, self-esteem, social relationships, and quality of life.

## Figures and Tables

**Figure 3:**